# On-Chip Purification of Tetracyclines Based on Copper Ions Interaction

**DOI:** 10.3390/s21217236

**Published:** 2021-10-30

**Authors:** Lorenzo Lunelli, Martina Germanis, Lia Vanzetti, Roberta Tatti, Cristina Potrich, Cecilia Pederzolli

**Affiliations:** 1 Fondazione Bruno Kessler, Center for Sensors and Devices, Via Sommarive 18, Povo, I-38123 Trento, Italy; lunelli@fbk.eu (L.L.); martina.germanis@gmail.com (M.G.); vanzetti@fbk.eu (L.V.); roberta.tatti@polito.it (R.T.); pederzo@fbk.eu (C.P.); 2 CNR—Consiglio Nazionale delle Ricerche, Istituto di Biofisica, Via alla Cascata 56/C, Povo, I-38123 Trento, Italy

**Keywords:** tetracycline purification, copper ions, polymeric microdevice, magnetic microbeads, food safety

## Abstract

Antibiotics are widely used to both prevent and treat bacterial diseases as well as promote animal growth. This massive use leads to the presence of residual antibiotics in food with severe consequences for human health. Limitations and regulations on the tolerated amount of antibiotics in food have been introduced and analytical methods have been developed. The bioanalytical methods usually employed to detect antibiotic residues, however, are time-consuming, expensive and laboratory-based. Novel methods with improved rapidity, portability and cost that are easy-to-use and sustainable are therefore highly desirable. In the attempt to fulfill this need, a microfluidic system was set up herein for the purification and pre-concentration of tetracyclines from raw milk selected as the case-study. The system includes a polymeric microfluidic chip containing magnetic beads loaded with copper to exploit the preferential interaction of tetracycline with divalent ions. The microfluidic system was demonstrated to efficiently pre-concentrate tetracycline, oxytetracycline and chlortetracycline with similar performances and efficiently purify tetracycline from raw milk without any pre-treatment. The simplified method described in this paper could be easily integrated in a compact and portable device for the in-field detection of tetracyclines, with the economic advantage of preventing food wastes and guaranteeing food safety.

## 1. Introduction

Since their discovery, antibiotics have principally been used to treat or prevent human and animal bacterial diseases. Later on, antibiotics were found to be able to improve growth and feed efficiency in food animals, leading to their widespread use as feed supplements with the side effect of their overuse in food-producing animals. As a consequence, the presence of antibiotic residues in food intended for human consumption has quickly become a critical issue for animal and human health (and for the environment as a whole)—in terms of both the possible direct toxicity and the development of antibiotic-resistant bacteria [1,2,3]. Therefore, reducing antibiotics use in livestock farming as well as in human medicine is of paramount importance for public health.

The need to ensure food safety has pushed national and international regulatory bodies to establish standards, guidelines and regulations [4,5]. The need to develop analytical methods to satisfy regulatory standards grew in parallel, and resulted in setting up protocols and techniques to rapidly and accurately detect, quantify, and confirm antibiotic residues in food. Recent methods include liquid chromatography with ultraviolet detection and mass spectrophotometry [6,7,8]; however, the use of portable rapid tests for on-site use or rapid screening methods is also growing [9,10]. The development of portable rapid tests is of great importance to prevent contaminated products from being processed and then placed on the market. Portable devices could indeed operate on site, i.e., where food raw materials are produced with non-negligible economical advantages.

In the context of field analyses, a microfluidic system was set up herein for the purification and concentration of antibiotics from food matrices, selecting tetracyclines and milk as the case-study. Tetracyclines are a family of antibiotics that inhibit protein synthesis by preventing the attachment of aminoacyl-tRNA to the ribosomal acceptor (A) site. Tetracyclines are broad-spectrum antibiotics, commonly used in veterinary medicine to prevent diseases and as an additive in animal foods to promote growth [11,12]. Since they are low-cost, well-adsorbed and rarely toxic drugs, their use has been extremely extensive over the last half century. This wide diffusion has lead not only to their undesirable accumulation in the environment [13] but also to the development of tetracycline-resistant bacteria [12,14]. In the attempt to contain this phenomenon, the Food and Agricultural Organization and the World Health Organization (FAO/WHO) and the European Union (EU) have indicated the maximum amount of tetracyclines permitted in food, named a maximum residue limit or MRL. The recommended MRL is 100 µg/kg (or ~0.1 ng/µL) for tetracyclines in milk [15].

Two main steps are needed to precisely quantify antibiotics in food matrices, i.e., sample preparation and antibiotic detection. The detection step is usually performed in the laboratory with chromatographic methods, while sample preparation comprises the isolation and/or the pre-concentration of compounds of interest from matrices, making the analytes more suitable for separation and detection. This step typically takes more than 70% of the total analysis time and regularly includes liquid–liquid extraction and solid-phase extraction [9]. Therefore, shortening and simplifying the sample preparation step whilst maintaining similar purification performances, will result in a clear improvement of the overall bioanalytical protocol. Starting from a polymeric microfluidic chip and magnetic beads, a purification/pre-concentration method based on a peculiar chemical property of tetracyclines, i.e., their interaction with metal ions, in particular with copper [16], was set up. Magnetic beads loaded with copper were inserted in a microfluidic chamber and fluxed with tetracycline spiked in buffer solution or in raw milk, without any pre-treatment. Fractions of tetracyclines were then eluted from the beads and quantified via high-performance liquid chromatography. The microfluidic system described herein was demonstrated as an efficient tool for the purification and concentration of various antibiotics belonging to the tetracyclines’ class, i.e., tetracycline, oxytetracycline and chlortetracycline. The three drugs were purified with very similar performances, validating the purification system for this class of antibiotics. Moreover, the system was demonstrated to efficiently purify tetracycline from raw milk without previous treatments. All the steps in the set-up of the purification system were fully characterized starting with the chemical composition of the beads to the type of beads and microfluidic chamber more suitable for this protocol. The encouraging results obtained in this work suggest the real possibility of developing portable rapid tests for on-site use, which potentially combine a simple on-chip sample preparation protocol with innovative methods of detection. A simplified and sensitive method of analyzing tetracyclines based on microfluidics, which could also be portable, could indeed overcome the limitations of protocols conducted under laboratory conditions as well as other very impactful limitations among which are those implied by the time needed to bring samples to where the analysis is performed and process them or the cost of instrumentation and skilled personnel.

## 2. Materials and Methods

### 2.1. Materials

Two types of magnetic beads were tested in this work: PureCube Cu-NTA MagBeads (Cube Biotech, Monheim am Rhein, Germany 25% suspension) and Dynabeads™ His-Tag Isolation & Pulldown (Thermo Fisher Scientific, Waltham, MA, United States, 40 mg beads/mL solution), endowed with copper and cobalt ions, respectively. Both particle types take advantage of nitrilotriacetic acid (NTA) chemistry to bind metal ions. The following reagents were purchased from Sigma-Aldrich: methanol, acetonitrile, oxalic acid, EDTA, copper sulfate, DPBS and all powders for buffer solutions. The Istituto Zooprofilattico Sperimentale del Piemonte, Liguria e Valle d’Aosta (IZSPLV), provided the raw milk tested for the absence of antibiotics and pure antibiotics for spiking solutions (tetracycline, oxytetracycline, chlortetracycline).

All microfluidic chips used in this work were purchased from the microfluidic ChipShop: four chambers, 100 µL volume each (Rhombic Chamber Chip eP1, Fluidic Design 221, PMMA, 600 µm depth); two chambers, 250 µL volume each (Rhombic Chamber Chip eP1, Fluidic Design 194, PMMA, 800 µm depth).

### 2.2. Characterization of Microbeads

The microparticles employed in this work were characterized in terms of size and chemistry. The size was checked under the microscope Leica DMLA (Leica Microsystems, Wetzlar, Germany). Dynabeads and Cu-NTA MagBeads were deposited on a microscope slide and observed with a 100 × and 20 × magnification objective, respectively. Images were acquired with a cooled CCD camera (DFC 420C, Leica Microsystems, Germany) and analyzed with the Fiji software [17].

The surface chemistry of the beads under different conditions was measured by X-ray photoelectron spectroscopy (XPS). Fifteen microliters (15 µL) of microparticles resuspended in pure water were deposited on 1 × 1 cm${}^{2}$ substrates of thermally grown silicon oxide, previously treated with argon plasma (10.5 W, 2 mbar, 1 min) to favor the beads’ adhesion. Samples were dried overnight at room temperature before measuring. Three samples of beads were prepared: (i) beads collected from the original solution; (ii) beads treated to remove the metal ions (cobalt or copper, depending on bead type); and (iii) beads re-loaded with copper ions. The removal of ions was performed as follows: 30 µL of original beads in a new vial were put on a magnetic rack (Dynabeads™ MPC™-S Magnetic Particle Concentrator) and supernatant was removed and substituted with 50 µL of EDTA at 100 mM and pH 8.3 for five removal cycles. After three further washing cycles with pure water, beads were suspended in 30 µL of pure water and equally divided into two fractions: the first fraction was deposited on silicon oxide, while the second was suspended in 150 µL of copper sulfate at 100 mM and incubated for 5 min at room temperature. After removing the supernatant, three more washing cycles with water were preformed before depositing the beads on the silicon oxide substrate.

XPS measurements were performed using a Scienta ESCA 200 instrument equipped with a hemispherical analyzer and a monochromatic Al Kα (1486.6 eV) X-ray source. The emission angle between the analyzer axis and the sample surface was 90°, corresponding to a sampling depth of approximately 10 nm. For each sample, Cu 2p, Co 2p, N 1s, O 1s and C 1s core lines were recorded at a pass energy of 150 eV, using a low electron energy flood gun to compensate sample charging. The quantification, reported as relative elemental percentage, was made using the integrated area of the core lines after the Shirley background subtraction [18] and using atomic sensitivity factors. XPS data were analyzed using the software described in Speranza and Canteri [19].

For Cu-NTA beads, all fractions collected during the protocol for copper removal and re-charging described above were measured by spectophotometer (Jasco V-550) to check the amount of copper present. Spectra from 500 to 900 nm were collected for washes with the EDTA solution and for the incubation with copper sulfate. The amount of copper present in the different fractions was quantified with suitable titration curves, prepared by measuring known amounts of copper in either EDTA solution or in water.

### 2.3. Purification System and Working Protocol

**Figure 1:**
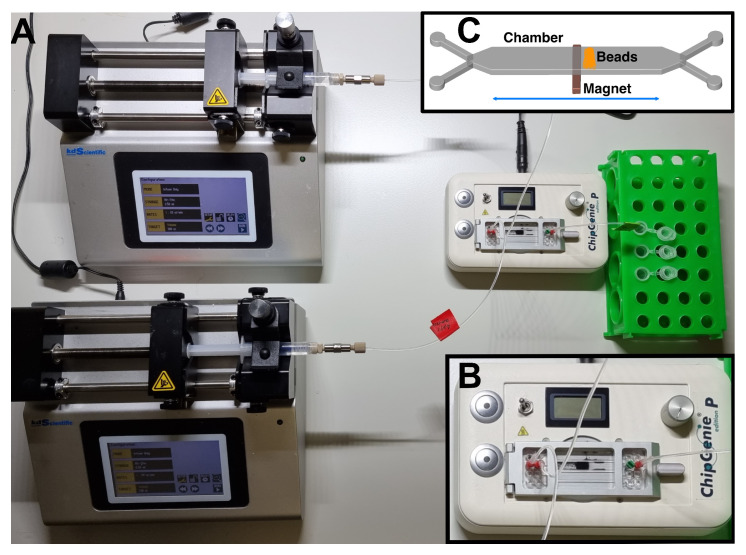
The purification system. Panel (**A**): two motorized syringes are feeding 4-chamber PMMA chips mounted on a “ChipGenie P” device (magnification in panel (**B**)) where a magnet can move the microbeads present inside the chambers (scheme of working principle in panel (**C**)).

**Figure 2:**
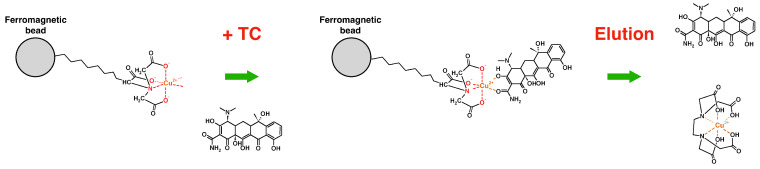
Scheme of the purification principle. Tetracycline binds the two free coordination sites of copper ions present on the surface of magnetic beads. When an acidic buffer containing EDTA is added, copper ions coordinate the EDTA molecules and tetracycline is eluted.

The system for the purification of tetracyclines was assembled starting from microfluidic PMMA chips mounted on the ChipGenie^®^ edition P device (microfluidic ChipShop). Fluids were injected in the chip inlets with two disposable syringes (Norm-Ject^®^, Sigma-Aldrich), actuated by two microsyringes (Legato 185, KD Scientific, Holliston, MA, USA) connected with fluoropolymer tubes (internal diameter 0.25 mm) and adapters (microfluidic ChipShop) (Figure 1A). The chip chambers were filled with magnetic beads (typically 30 µL diluted in 350 µL of DPBS for both type of beads), which were actuated with the ChipGenie^®^ edition P device during the experiment (Figure 1B,C). The beads exposed copper ions on their surface, which were able to bind tetracyclines. Tetracyclines were then eluted by an EDTA-based buffer (McIlvaine B: 0.1 mM EDTA, 61.4 mM citric acid, 77 mM Na${}_{2}$ HPO${}_{4}$ pH 4) which coordinate the copper ions, releasing the antibiotic (Figure 2). Dynabeads are indeed functionalized with highly specific IMAC chemistry. The technology is comprised of a tetradentate metal chelator in which four of copper’s six coordination sites are occupied. The tetracycline molecule is able to occupy the two remaining coordination sites, resulting in antibiotic binding. Tetracycline indeed possesses multiple electron-donor functional groups, which are stabilized by complexing with highly polarizing transition metals. The positive charge of the metals is partially shared with the negative charge of electron donor atoms present in tetracycline and electron delocalization over the sharing atoms (chelating ring) which stabilizes the large electron density of tetracycline [20]. Once captured, tetracycline is released only when a molecule, such as EDTA, is substituted to tetracycline under acidic pH conditions and copper ions are removed from the metal chelator groups on the beads (Figure 2). The same working principle is shared by Cu-NTA beads which are made of agarose. In this case, an NTA ligand is coupled to the agarose of Cu-NTA beads and loaded with copper ions to obtain a matrix with the highest binding capacity.

A typical protocol for tetracycline purification was performed as follows: one chamber of the microfluidic chip was first fluxed with 400 µL of DPBS with a flux of 100 µL/min through the first chamber inlet, while in the second inlet, 350 µL of beads (“as-received” as well as “re-loaded” beads) diluted in DPBS were injected with a micropipette. The device magnet motor was kept off during beads injection. Then, 200 µL of DPBS at a speed of 50 µL/min were fluxed to remove beads which were possibly not firmly attracted by the magnet. An excess of solution (either DPBS or raw milk) was spiked with the antibiotic at the desired concentration (e.g., 0.1 ng/µL in 2.5 mL solution) and injected at a speed of 50 µL/min into the second inlet. After washing with 400 µL of DPBS at 20 µL/min, the antibiotic was finally eluted with 420 µL of McIlvaine B buffer at 20 µL/min. A washing step with 200 µL DPBS at 50 µL/min was finally performed. All fractions were collected from the outlet for the quantification via HPLC, while the used beads remained in the chamber and were discarded together with the disposable chip.

### 2.4. Analysis of Fractions Collected with the Purification Device

The amount of tetracycline present in the fractions collected at the outlet of the chip chamber was quantified by HPLC. The instrument employed was a Shimadzu composed of the following modules: detector Shimadzu prominence UV/Vis Detector SPD-20A, Shimadzu prominence Communications Bus Module CBM-20A, Shimadzu Liquid Chromatograph LC-20AB. A degasser (Waters™ In-Line Degasser) was also mounted to improve HPLC performances, while fractions were manually injected through a Rheodyne^®^ model 7725i injector connected with a 50 µL loop. Data were acquired with the LCSolution software (Shimadzu). An inverse phase column was used for separations (Luna^®^ C18 by Phenomenex^®^, 5 µm, 100 Å, 250 mm × 3 mm connected with the pre-column Security Guard™ by Phenomenex^®^). All fractions were centrifuged at 21,000× *g* for 10 min at room temperature, before injection in HPLC, to possibly present pellet beads. A mobile phase was composed of solvent A (oxalic acid 0.01 M pH 2.7) and solvent B (methanol and acetonitrile in a 1.5:1 ratio), pumped with a 0.35 mL/min flux. The separation method was the following: 10% solvent B for 5 min, gradient up to 58% of solvent B in 20 min.

## 3. Results and Discussion

The set-up of the on-chip purification and concentration of tetracyclines started with the testing of the microfluidic system in terms of volumes, chip chambers and types of beads carrying copper ions on their functional surface. The performances of the system were tested with chips of different volume and containing two types of different beads. Then, different amounts of tetracycline spiked in buffer and other antibiotics were purified with the system before testing the purification of tetracycline in raw milk.

The microfluidic system and chips were selected from commercially available tools to allow the rapid development of the purification method without waiting for system design and prototyping, which was left to a future phase, when it will be possible to directly fabricate specific tools optimized with respect to already commercially available devices. Moreover, the possibility to build up a microfluidic system from off-the-shelf parts allowed to explore the potentialities of microfluidics with respect to bench activity.

### 3.1. Characterization of Magnetic Beads

**Figure 3:**
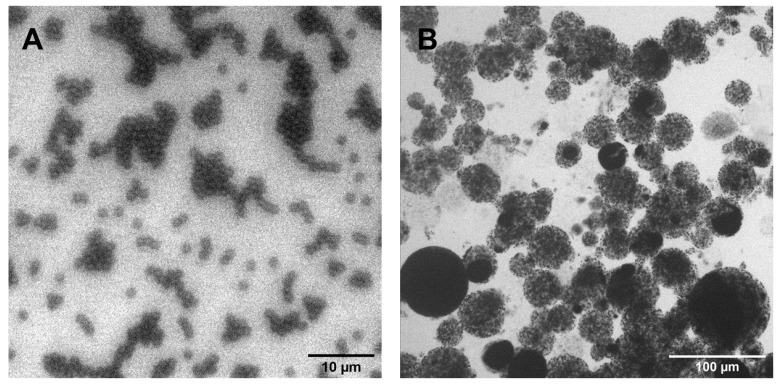
Microscope images of magnetic beads: a 10-fold diluted solution of Dynabeads ((**A**); scale bar 10 µm) and a solution 2-fold diluted solution of Cu-NTA ((**B**); scale bar 100 µm).

Two types of beads with different size and functionality were selected for capturing tetracyclines: Dynabeads (1 µm mean diameter, exposing Co${}^{2+}$ on the surface) and Cu-NTA (30 µm average diameter, exposing Cu${}^{2+}$). These beads were also selected for their different surface-to-volume ratio in order to verify the most efficient combination for the purification of tetracyclines. Optical microscope images (Figure 3) confirmed the dimension of the beads, with Dynabeads showing a very narrow distribution peaked at 1.4 µm, while Cu-NTA showed a polydispersed distribution ranging from small beads (diameter of approximately 15 µm) up to more than 30 µm diameter beads due to their different composition and synthesis methods. The magnetic core of the Dynabeads is indeed coated with polystyrene, functionalized to expose NTA groups, which coordinate divalent ions (as declared by the manufacturer), while Cu-NTA beads are prepared from agarose, which entraps more magnetic cores, resulting in a population of beads with a different diameter, as visible in Figure 3.

In addition to morphology, the surface chemistry of both types of beads under different conditions was characterized by XPS analysis. Beads were measured as received from the producer (named “new” in Table 1) after removing the metal ion (cobalt for Dynabeads and copper for Cu-NTA) with the protocol described in Section 2.2 (named “dis-charged” in the same table) and then re-loaded with copper sulfate (named “re-loaded”). The protocol used for the removal of ions was fully efficient in removing both cobalt and copper ions, confirming that EDTA is a good candidate for the elution of molecules coordinated by divalent ions. For both types of beads, no cobalt nor copper was measured after the dis-charging treatment, while the subsequent treatment of reloading with copper ions resulted in an amount of copper similar to the original ion surface percentage for Cu-NTA beads and slightly lower for Dynabeads. The characterization of beads reloaded with copper demonstrated the possibility of substituting cobalt with copper for Dynabeads and the amount of copper ions on CuNTA beads was the maximum one possible, i.e., no more copper ions could be adsorbed by Cu-NTA. The percentage of nitrogen measured with XPS (Table 1) was instead constant, as expected for the presence of -NTA groups, covalently bound to the surface of both bead types. In addition, carbon and oxygen percentages were also almost constant for the three samples of the same beads type, either Dynabeads or Cu-NTA. As mentioned above, Dynabeads present polystyrene on their surface and -NTA groups bound to polystyrene with a proprietary chemistry. The -NTA group contains six oxygen atoms (while polystyrene does not contain oxygen) and one nitrogen atom, therefore the ratio of approximately 4–5 oxygen per nitrogen found for Dynabeads is reasonably consistent with the structure of this molecule. Furthermore, in the case of Cu-NTA, carbon and oxygen contents are constant for the three samples measured, with a ratio carbon/oxygen of approximately 0.7. In this case, the amount of oxygen is higher than that for Dynabeads, since Cu-NTA are made of agarose, a polymer composed of carbon and oxygen.

In addition to the testing of the quality of beads in terms of the presence of copper due to different treatments, the regenerability/re-usability of Cu-NTA was also analyzed via XPS. A fraction of beads was indeed measured after capturing and eluting tetracycline, finding no copper present after the elution step. The same beads were re-loaded with copper ions, which resulted in a similar amount as the original commercial beads (data not shown). In principle, beads could be regenerated and re-used several times, but this step is quite laborious and it was therefore considered more economically convenient to use new beads for every experiment.

### 3.2. Selection of the Optimal Chip Chamber/Volume and Beads

**Figure 4:**
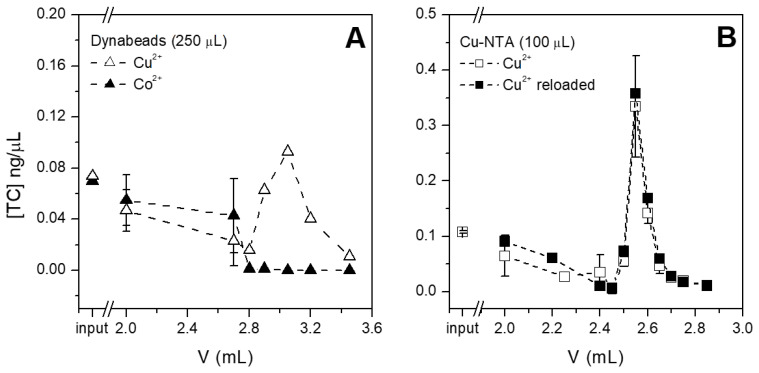
Tetracycline capture and release from (**A**) Dynabeads loaded in the 250 µL chambers without further treatment, i.e., exposing Co${}^{2+}$ (black triangles) or re-loaded with Cu${}^{2+}$(white triangles); and (**B**) Cu-NTA beads in 100 µL chambers without treatments (Cu${}^{2+}$, white squares) or reloaded (black squares). Error bars represent the standard deviation.

The performances of both magnetic beads (Dynabeads and Cu-NTA, new and re-loaded with the protocol described in Section 2.2) were tested in terms of the capture and release of tetracycline spiked in buffer. The beads were inserted in the microfluidic devices, fluxed with tetracycline solutions, then with washing buffer and finally with the elution buffer (see Section 2.3), while fractions were collected after every step. The amount of tetracycline present in every fraction was quantified via HPLC and the concentration was plotted versus the volume fluxed in the microfluidic device, as shown in Figure 4. The concentration of tetracycline was selected as the most informative parameter to express the performances of the purification system, since not only the purification of antibiotics but also their pre-concentration are both crucial parameters in the optics of portable tests, which at present are suffering from rather scarce sensitivity. Dynabeads reloaded with copper captured and eluted a good amount of tetracycline, which was concentrated at approximately 25% in the highest eluted fraction (Figure 4A). On the contrary, Dynabeads exposing Co${}^{2+}$ on the surface i.e., as received from the producer, poorly captured tetracycline and no release was detectable. In this case, more than 95% of injected tetracycline was indeed found in the unbound solution and in the washes performed before elution. Even if copper and cobalt can be bound to the same site of tetracycline [20], the efficacy of this interaction is greatly influenced by the pH of the solution and by the respective concentration of tetracycline and metal ions. Since the use of cobalt-carrying beads led to tetracycline mostly being found in the unbound and washes fractions (i.e., in the first 2.7 mL fluxed in the chamber, as can be seen in Figure 4A), this antibiotic may not interact or may poorly interact with cobalt. It should be noted that the selected amount of copper-loaded beads can instead capture more than 26% of the injected tetracycline. This amount could be considered as not significant for purification; however, this experiment is aimed not at capturing all the antibiotic present in the original sample, but at concentrating the purified antibiotic and removing the original matrix.

In addition to Dynabeads, Cu-NTA beads were also tested for their capture and release of tetracycline (Figure 4B) under the “new” and “reloaded” conditions (see previous paragraph), but finding no significant difference. This result confirms the XPS data, where the amount of copper measured under these two conditions was similar, indicating that Cu-NTA beads already contain the maximum concentration of copper ions when new. Therefore, Cu-NTA beads were used without further treatment in all subsequent experiments, while Dynabeads were used after the substitution of previously loaded cobalt with copper ions.

**Figure 5:**
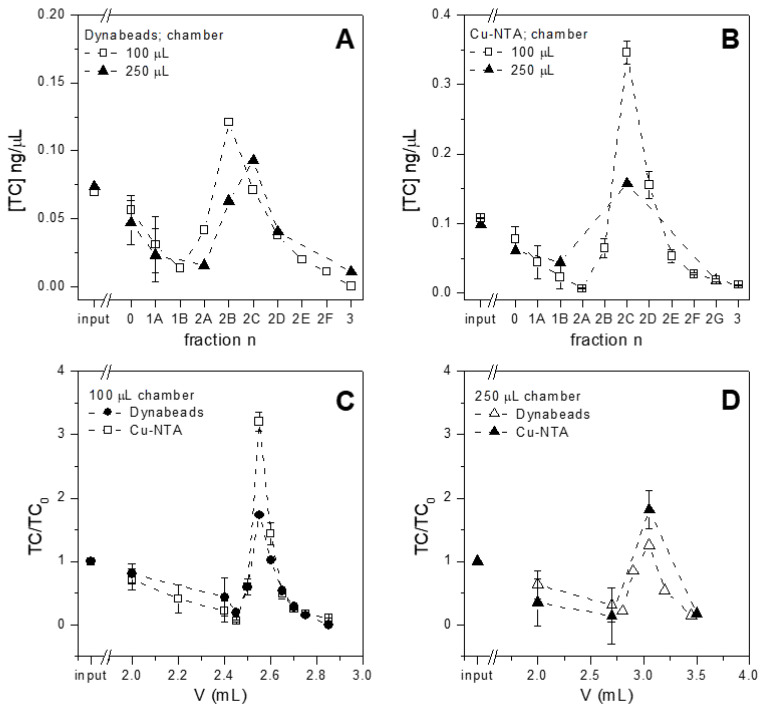
Purification of tetracycline spiked in buffer with microfluidic chips carrying chambers of different volumes and loaded with the two types of beads. Panel (**A**): Dynabeads tested in different chambers; panel (**B**): Cu-NTA beads in different chambers. The x axis shows the fractions collected at the outlet of the system, and in particular, fraction 0 represents the tetracycline collected during injection (unbound), fractions 1A–1B represent the volume collected during washes, while fractions 2A–2G represent the volume collected during the elution step and finally, fraction 3 represents a final wash; Panels (**C**,**D**): the tetracycline concentration found in different fractions was normalized to the concentration of injected tetracycline (TC/TC${}_{0}$) in same volume chambers (100 µL in panel (**C**) and 250 µL in panel (**D**)) loaded with the two types of beads. The x axis shows the volume fluxed in the microfluidic chambers.

Both types of magnetic beads were tested in microfluidic chambers of different volumes for the purification of tetracycline spiked in buffer (Figure 5). When Dynabeads were used (Figure 5A), a higher amount of tetracycline was captured and released from the beads loaded in the 100 µL chamber with respect to the higher volume chamber (250 µL). Similar results were obtained for Cu-NTA beads which show the best performances with 100 µL chamber (Figure 5B). Therefore, the 100 µL chamber seems the most promising volume under these experimental conditions. The 250 µL chamber needs larger loading volumes and longer times of analysis, even if the overall performances seem good. In addition to chamber volume, the performances of the two types of beads were compared. Cu-NTA beads show better elution ability than Dynabeads, especially when both are loaded in the 100 µL chamber (Figure 5C). They are indeed able to concentrate the tetracycline by more than 3 times with respect to the initial antibiotic concentration, while Dynabeads under the same conditions reached a less than 2-fold value. For the 250 µL chamber, analogous results were obtained (Figure 5D), even if Dynabeads could concentrate the tetracycline by a 1.5-fold factor, while Cu-NTA could concentrate the tetracycline by a 2-fold factor. A possible explanation of the better performances observed by using Cu-NTA beads could that of the intrinsic properties of the beads. Dynabeads are indeed much smaller and have stronger magnetic properties compared to Cu-NTA beads and therefore are firmly held by the magnet during the experiment, with the final result of the distribution spread on the lower part of the chamber. The tetracycline solution as well as the elution buffer could in this case pass over and not through the beads. This hypothesis is supported by experiments performed with the same amount of tetracycline and Dynabeads in the vial, following the manufacturer’s instructions; these experiments gave an optimal recovery of tetracycline (data not shown).

The results presented in this section indicate the 100 µL chamber and Cu-NTA beads as the most promising combination for subsequent experiments.

### 3.3. Testing the System with Different Amounts and Types of Antibiotics

**Figure 6:**
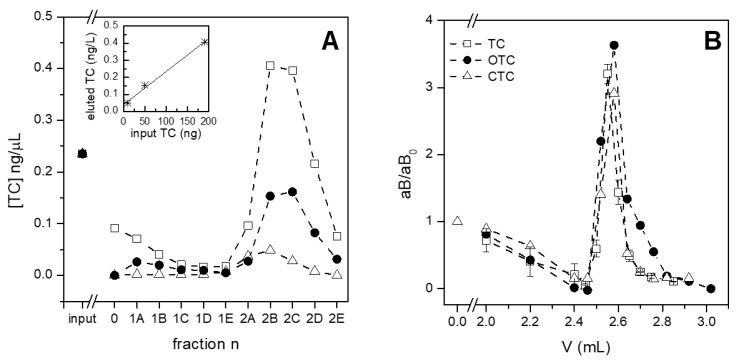
Test of the purification system with different amounts of tetracycline (panel (**A**)) and different tetracyclines (panel (**B**)). A: the same concentration of tetracycline dissolved in DPBS was injected in the 250 µL chamber containing the same amount of Cu-NTA beads for different times (white square: 16 min; solid circles: 4 min and white triangles: 1 min). Then, the chamber was fluxed with DPBS (fractions 1A–1E) and finally tetracycline was eluted with the McIlvaine buffer (fractions 2A–2E). The amount of tetracycline was quantified by HPLC. Inset: linear correlation between input and eluted tetracycline. B: Cu-NTA beads were loaded in 100 µL chambers, where the different antibiotics spiked in buffer were purified and concentrated. The reported values “aB/aB${}_{0}$” refer to the antibiotic present in fractions normalized for the injected antibiotic.

Once the best performing beads and chambers were selected, the subsequent experiments were devoted to evaluating the efficacy of the purification system. The dynamic range of tetracycline capture was explored by injecting a solution of antibiotics for different durations of time. Starting from the amount of tetracyclyne injected in previously presented experiments (Figure 4 and Figure 5), i.e., approximately 200 ng, lower quantities were tested. A constant concentration of tetracycline spiked in buffer (i.e., 0.23 ng/µL) was indeed flowed on the same amount of Cu-NTA beads (i.e., 30 µL) in three parallel chambers (Figure 6A). The three amounts of tetracycline were differently captured by Cu-NTA beads. When 50 µL of solution flowed into the chamber, a total amount of 12 ng of tetracycline was injected and completely captured, as evidence by the quantification of the antibiotic present in the collected fractions corresponding to unbound and washes, which did not contain a significant amount of tetracycline (white triangles, fractions 0 and 1A–1E, Figure 6A). On the contrary, when the chip was loaded with 47 ng and 190 ng of tetracycline, 85% and 60% of the antibiotic was adsorbed, respectively. Under these conditions, Cu-NTA beads loaded in the chamber were not sufficient for capturing all the tetracycline injected, as evidenced for the amount of tetracycline found in fractions corresponding to unbound material and first washes (fraction 0 and 1A and B, respectively, in Figure 6A). Moreover, the injection of 190 ng of tetracycline resulted in the elution of more concentrated fractions, while the injection of lower amounts produced fractions more diluted than the original sample. In addition to the concentration, a similar behavior was instead observed for the release of tetracycline, i.e., all the captured tetracycline was eluted under all the tested conditions. A concentration factor was also evident in the eluted fractions, especially for the highest amount of loaded tetracycline. In fact, the more tetracycline is inserted in the chamber, the more is eluted, with a linear correlation (Figure 6A, inset). It should be noted that the minimal amount of tetracycline to be captured according to current regulations, i.e., 100 µg/Kg or, approximately, 0.1 ng/µL, corresponds to a total of 25 ng, if one volume of the chamber is inserted, in line with the performance of the purification system loaded with 30 µL of CuNTA beads. Moreover, these results indicate that the purification system could be efficiently operated in two modes: (i) in static mode by filling the chamber, waiting for a few minutes for a suitable interaction of tetracycline with copper on beads and then eluting the antibiotic; and (ii) in dynamic mode by continuously fluxing the sample, as in this experiment, which is particularly useful for the capture of antibiotics in diluted samples. The main limitations of the portable tests on the market are the scarce sensitivity and the quite high limit of detection. A pre-purification step which is also able to concentrate the analytes could add a significant value to these tests; therefore, the longest injection time was selected for all the subsequent experiments.

In addition to the amount of tetracycline, two other antibiotics belonging to the tetracycline family, i.e., oxytetracycline and chlortetracycline, were tested under the same conditions as tetracycline (Figure 6B). The three antibiotics were spiked in buffer under the same conditions and all were captured and eluted with similar yields, resulting in approximately 3.5-fold more concentrated fractions. Both purification and concentration are crucial parameters for preparing suitable samples for subsequent analysis—especially for label-free detection. In the optic of an integrated device, the possibility to process a raw material such as milk, extracting and concentrating even minimal amounts of antibiotics, leads to potential in-field analyses, and saving time and money.

### 3.4. Purification of Tetracycline from Raw Milk

**Figure 7:**
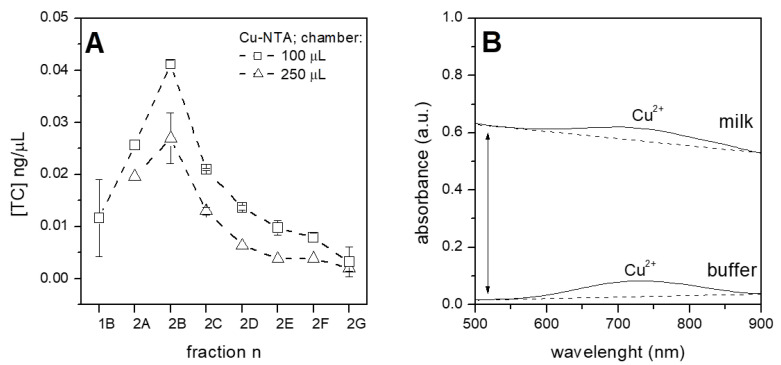
Purification of tetracycline spiked in raw milk. Panel (**A**): Cu-NTA beads were loaded either in 100 µL chamber (squares) or in 250 µL chamber (triangles) and tetracycline was purified with the microfluidic system; and Panel (**B**): spectroscopic analysis of the eluted fractions, starting from tetracycline spiked in buffer (lane named “buffer”) or in raw milk (lane “milk”). Peaks evidence the presence of copper ions (similar for both samples, as evidenced from the dotted lines). Absorbance was normalized to allow the comparison of samples.

The potentialities of the purification system described in this paper were tested with raw milk and certified for the absence of antibiotics. Tetracycline at a concentration of 0.1 ng/µL was spiked in milk, processed with the system, and the collected fractions were quantified by HPLC as usual (Figure 7A). Both tested chip chambers gave the same profile of purification with the 100 µL chamber performing slightly better, as seen in all other experiments. The possible presence of the milk matrix was investigated with spectroscopic measurements (Figure 7B), observing a negligible amount of material still present after elution and quantifiable as less than 0.6% with respect to the initial milk value (this value was estimated by comparison with a titration curve, measured separately). This amount is perfectly compatible with the following analyses, as evident from HPLC, since the eluted fractions were clear enough to permit HPLC quantification. Moreover, the amount of copper ions, which are eluted together with tetracycline (indicated by the areas under peaks in the spectra shown in Figure 6B), was similar for the buffer and milk samples, indicating that the elution step is equally efficient and that the reduced yield of tetracycline purified from milk (compare Figure 5B and Figure 7A) is due to a reduced capture of antibiotic rather than to a retention of tetracycline on beads. As evidenced in Figure 6A, no concentration of tetracycline was observed in this experiment. This phenomenon could be due to the different matrix molecules present in raw milk, such as proteins, fat or ions. Milk is indeed a complex fluid, containing among others huge amount of divalent ions which could possibly interfere with copper-based purification. Calcium is particularly abundant as it is the free calcium ion present in a millimolar concentration [21]—possibly high enough to compete with copper ions for the binding of tetracyclines.

**Figure 8:**
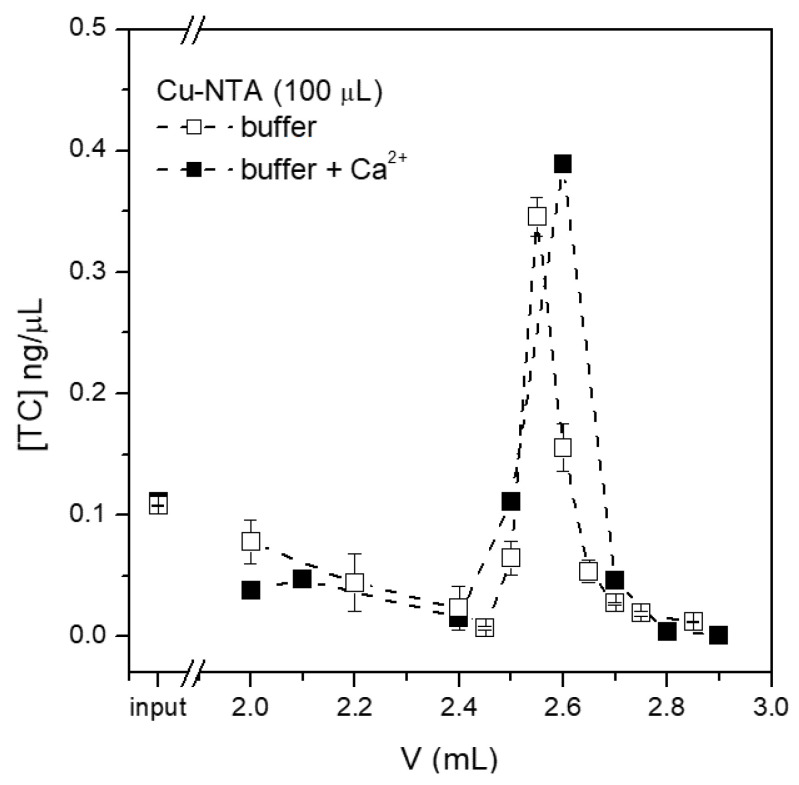
Effect of calcium ions on the purification of tetracycline. Chip chambers of 100 µL loaded with same amount of Cu-NTA beads were employed.

To elucidate the role of calcium ions for the purification of tetracycline, a sample containing 3 mM Ca${}^{2+}$ in buffer, in addition to the antibiotic, was prepared and injected into the purification system. The comparison between the presence and absence of calcium ions (Figure 8), allowed us to conclude that calcium does not compete with copper for tetracycline binding and purification in the presence of calcium is similarly efficient to that when calcium is absent. Indeed, calcium ions are known to bind to a different site of the tetracycline molecule with respect to copper [20] and therefore, even when tetracycline is mixed with a high amount of calcium ions, the binding site for copper ions is free for the capture of magnetic beads. In conclusion, the reduction in tetracycline capture by Cu-NTA beads from milk is not due to calcium ions, but other molecules could non-specifically coat copper binding sites, impeding the concentration of tetracycline from raw milk.

The microfluidic system presented herein efficiently purifies tetracycline from raw milk, however, the purification yield is less optimized with respect to a simplified matrix such as phosphate buffer. However, all commercial tests for the quantification of antibiotics in food require sample pre-treatment to remove most of matrix contaminants which interfere with the measure. The system described in this paper instead does not need any dilution or treatment such as defatting prior to being processed, allowing one to save time and money and simplifying the overall purification protocol. These benefits could compensate for the lack of concentration, especially with the view of portable devices.

## 4. Conclusions

The wide use of antibiotics for both treating diseases and promoting animal growth also led to their presence in food intended for human consumption, with non-negligible issues for human health. Authorities established regulations in order to contain this problem, fixing a maximum amount of antibiotics possibly present in food. In parallel, new protocols and technologies have emerged for quantifying the presence of antibiotics in a reliable and precise way though with variable results. Here, a new method based on a microfluidic chip combined with copper-coated magnetic beads is proposed in order to simplify the overall procedure and making a step forward toward portable, compact and easy-to-use devices for the in-field control of antibiotics in food. Tetracyclines and milk were selected as the case-study, while off-the-shelves technology was assembled in order to set up a purification system. The possibility to build up a microfluidic system from commercially available tools allowed to explore the potentialities of microfluidics with respect to bench activity and bring the analysis of antibiotics into the field and out of laboratories. The system was tested with different microfluidic chambers and magnetic beads and the best performing parameters were applied for the successful purification of different antibiotics belonging to the tetracyclines class in simplified or complex matrices. The on-chip purification of tetracycline from raw milk resulted in clear fractions, suitable for subsequent analyses, without the need for previous treatments. The simplified method described in this paper could be easily integrated in a compact device for the field detection of tetracyclines with the economic advantage of rapid on-site analysis, which allows removing raw aliments from production before processing. In other words, a similar compact and portable device could have as final outcome the prevention of food wastes and the guarantee of food safety.

## Figures and Tables

**Table 1:** XPS analysis at 90° take-off angle of the two types of beads with different treatments. The standard error does not exceed the 1–2% of the reported value.

**Dynabeads**	**Co 2p (%)**	**Cu 2p (%)**	**N 1s (%)**	**O 1s (%)**	**C 1s (%)**
new	1.3	-	5.9	24.4	68.5
dis-charged	-	-	5.1	24.7	70.2
re-loaded	-	0.7	5.6	24.9	68.8
**Cu-NTA**	**Co 2p (%)**	**Cu 2p (%)**	**N 1s (%)**	**O 1s (%)**	**C 1s (%)**
new	-	0.5	1.4	49.3	34.9
dis-charged	-	-	2.8	46.6	38.2
re-loaded	-	0.4	1.5	49.2	35.2

## Abbreviations

The following abbreviations are used in this manuscript:

TC	tetracycline
MRL	maximum residue limit
NTA	nitrilotriacetic acid
EDTA	ethylenediaminetetraacetic acid
XPS	X-ray photoelectron spectroscopy
